# Molecular and Biological Characterization of the First Mymonavirus Identified in *Fusarium oxysporum*

**DOI:** 10.3389/fmicb.2022.870204

**Published:** 2022-04-21

**Authors:** Jing Wang, Chengjun Li, Pengyu Song, Rui Qiu, Ruifang Song, Xiaojie Li, Yunxia Ni, Hui Zhao, Hongyan Liu, Shujun Li

**Affiliations:** ^1^Key Laboratory for Green Preservation and Control of Tobacco Diseases and Pest in Huanghuai Growing Area, Institute of Tobacco, Henan Academy of Agricultural Sciences, Zhengzhou, China; ^2^College of Biological Sciences, China Agricultural University, Beijing, China; ^3^Tobacco Company of Henan Province, Zhengzhou, China; ^4^Key Laboratory of Integrated Pest Management on Crops in Southern Region of North China, Henan Key Laboratory of Crop Pest Control, Institute of Plant Protection, Henan Academy of Agricultural Sciences, Zhengzhou, China

**Keywords:** mycovirus, *Fusarium oxysporum*, *Mymonaviridae*, *Hubramonavirus*, root rots

## Abstract

We characterized a negative sense single-stranded RNA mycovirus, *Fusarium oxysporum* mymonavirus 1 (FoMyV1), isolated from the phytopathogenic fungus *Fusarium oxysporum*. The genome of FoMyV1 is 10,114 nt, including five open reading frames (ORFs1–5) that are non-overlapping and linearly arranged. The largest, ORF5, encodes a large polypeptide L containing a conserved regions corresponding to *Mononegavirales* RNA-dependent RNA polymerase and mRNA-capping enzyme region V; the putative functions of the remaining four ORFs are unknown. The L protein encoded by ORF5 shared a high amino acid identity of 65% with that of Hubei rhabdo-like virus 4, a mymonavirus that associated with arthropods. However, the L protein of FoMyV1 also showed amino acid similarity (27–36%) with proteins of mynonaviruses that infect the phytopathogenic fungi *Sclerotinia sclerotiorum* and *Botrytis cineaea*. Phylogenetic analysis based on L protein showed that FoMyV1 is clustered with the members of the genus *Hubramonavirus* in the family *Mymonaviridae*. Moreover, we found that FoMyV1 could successfully transfer by hyphal anastomosis to a virus-free strain. FoMyV1 reduced the vegetative growth and conidium production of its fungal host but did not alter its virulence. To the best of our knowledge, this is not only the first mymonavirus described in the species *F. oxysporum*, but also the first *Hubramonavirus* species found to infect a fungus. However, the incidence of FoMyV1 infections in the tested *F. oxysporum* strains was only 1%.

## Introduction

The genus *Fusarium* is a class of filamentous fungi that includes endophytes, saprophytes, and pathogens (Knogge, 1996). It is most notable for a devastating phytopathogenic fungus that causes severe losses in many economically important crops (Pietro et al., 2003; Michielse and Rep, 2010; Sharma et al., 2018). *Fusarium oxysporum*, generally regarded as a species complex, causes vascular wilt disease, damping-off, and crown or root rots (Farquhar and Peterson, 2010; Gordon, 2017). Frequent and intensive applications of fungicides have led to the emergence of resistant pathogen strains in fields (Chen et al., 2007; Xu et al., 2015), along with ecosystem destruction that threatens food security and human health (Fisher et al., 2012). Therefore, new biological control strategies for eco-friendly control of *F. oxysporum* are badly needed.

Mycoviruses are viruses that infect fungi which are ubiquitous across the kingdom Fungi (Ghabrial et al., 2015). Most known mycoviruses are composed of double-stranded RNA (dsRNA) genomes, about 30% have positive-sense single stranded (+)ssRNA genome, a few have negative-sense (−)ssRNA genome (Ghabrial et al., 2015), and an even smaller number have circular single-stranded DNA genome (Yu et al., 2010; Li et al., 2020; Hao et al., 2021). Mycovirus infections are often cryptic; in some cases, however, they induce hypovirulence in their fungus host (Ghabrial and Suzuki, 2009). An increasing number of hypovirulence-associated mycoviruses have been used as potential viral agents. For example, Cryphonectria hypovirus 1 (CHV1) has been successfully utilized to control the disastrous chestnut blight caused by *Cryphonectria parasitica* in Europe (Anagnostakis, 1982). Fungal virus infections can affect the fungicide sensitivity of the host. Co-infection of Penicillium digitatum polymycovirus 1 and Penicillium digitatum narna-like virus 1 can reduce the fungicide resistance of *Penicillium digitatum* (Niu et al., 2018). Persistent infection with Phytophthora endornavirus 2 and Phytophthora endornavirus 3 may impact the fungicide sensitivity of the host oomycete (Uchida et al., 2021). The well-studied Sclerotinia sclerotiorum hypovirulence-associated DNA virus 1 (SsHADV-1) can convert its host (*Sclerotinia sclerotiorum*) from a typical necrotrophic pathogen to a beneficial endophytic fungus (Yu et al., 2013; Zhang et al., 2020). SsHADV-1 and similar mycoviruses are sometimes referred to as “plant vaccines” because their application to crops represents a new and useful approach to disease control.

The evidence of (–)ssRNA virus may infect fungi in nature was first found in 2013 (Kondo et al., 2013). Sclerotinia sclerotiorum negative-strand RNA virus 1 (SsNARV-1), the first (–)ssRNA virus was obtained and characterized as infecting a fungus, belongs to the newly proposed family *Mymonaviridae*, order *Mononegavirales* (Liu et al., 2014; Jiāng et al., 2019). This family contains nine genera: *Auricularimonavirus*, *Botrytimonavirus*, *Hubramonavirus*, *Lentimonavirus*, *Penicillimonavirus*, *Phyllomonavirus*, *Plasmopamonavirus*, *Rhizomonavirus*, and *Sclerotimonavirus*. Five of these genera (the exceptions being *Hubramonavirus*, *Phyllomonavirus*, *Plasmopamonavirus*, and *Rhizomonavirus*) have been reported to infect fungi. The typical mymonavirus genome is predicted to have five or six major non-overlapping ORFs that expressed as individual transcription units and are separated by non-coding intergenic regions containing highly conserved gene junction sequences (Jiāng et al., 2019). One member of the *Mymonaviridae* is known to infect the genus *Fusarium*: Fusarium graminearum negative-stranded RNA virus 1 infects *F. graminearum* (Wang et al., 2018).

*Fusarium oxysporum* is an important pathogenic fungus on many economically important crops, causing Fusarium root rots. Several mycoviruses have been reported to infect this fungus, including four dsRNA mycoviruses, *Fusarium oxysporum* chrysovirus 1 (FoCV1, ICTV approved), *Fusarium oxysporum*f. sp. dianthi virus 1 (FodV1, ICTV approved), *Fusarium oxysporum* alternavirus 1 (FoAV1), and Hadaka virus 1 (HadV1), from the families *Chrysoviridae, Alternaviridae*, and *Polymycoviridae*, respectively (Sharzei et al., 2007; Lemus-Minor et al., 2015; Sato et al., 2020; Wen et al., 2021). Moreover, several (+)ssRNA viruses infect *F. oxysporum*, including *Fusarium oxysporum* ourmia-like virus 1 (FoOuLV1), *Fusarium oxysporum* f. sp. dianthi hypovirus 2 (FodHV2), and *Fusarium oxysporum* f. sp. dianthi mitovirus 1 (FodMV1), in the families *Botourmiaviridae*, *Hypoviridae*, and *Mitoviridae*, respectively (Torres-Trenas and Pérez-Artés, 2020; Torres-Trenas et al., 2020; Zhao et al., 2020; Wang et al., 2021). Also noteworthy is HadV1 has a potential novel lifestyle as a multisegmented RNA virus. Among these mycoviruses, FodHV2 does not alter the vegetative growth, conidiation, or virulence of its fungal host. However, FodV1 and FoOuLV1 showed significant biological control potential on Fusarium wilt.

In this study, we identified and characterized a novel (–)ssRNA mycovirus found in *F. oxysporum* strain LJ3-3, which we named *Fusarium oxysporum* mymonavirus 1 (FoMyV1). It belongs to the family *Mymonaviridae* and is the first mymonavirus identified in *F. oxysporum*. It is also the first virus in the genus *Hubramonavirus* reported to infect a fungus. Here, we describe its transmission ability and effects on its host.

## Materials and Methods

### Fungal Strains and Culture Conditions

The *Fusarium oxysporum* strain LJ3-3 used in this study was recovered in 2020 from a capsicum root rot sample (Luohe, Henan Province, China). The strain AJ3-8 of *F. oxysporum* was used as a control. The diseased root was cut into 0.5-cm^2^ samples and soaked for 30 s in 75% ethanol. Then, the samples were rinsed with sterilized water three times and dried on sterilized blotting paper. Finally, the samples were cultured on potato dextrose agar (PDA) medium at 25°C in the dark for 2 days. A small amount of mycelium was scraped off the culture and washed with 2 ml sterile water. Then, the mixture was pooled and filtered through three-layer lens wiping paper. The spore liquid was diluted to 10^3^ conidia ml^–1^, and 100 μl was smeared on a PDA plate and incubated overnight at 25°C. The next day, a single colony was selected and considered a purified strain. Five mycelial agar plugs were inoculated on fresh PDA medium covered with cellophane membranes and cultured at 25°C for 4–5 days. Mycelium in each dish were harvested and stored at –70°C until use. Genomic DNA was extracted from the fungi using the CTAB method. The primers for translation elongation factor 1-alpha (EF-1α), RNA polymerase II subunit I gene (RPB1), and RNA polymerase II subunit II gene (RPB2) were used to confirm the *Fusarium* species identification (Mishra et al., 2003; O’Donnell et al., 2010). The EF-1α, RPB1, and RPB2 sequences were analyzed by Blast search against data in the Fusarium ID: Cyber-infrastructure for Fusarium database (fusariumdb.org). Mycelial growth and conidial production were evaluated according to the procedures described by Wu et al. (2007). A transformant of *F. oxysporum* strain B9 was used as a recipient strain in a horizontal transmission test. The B9 strain was isolated from a fusarium root rot sample of tobacco (Xuchang, Henan Province, China, 2020). This B9 strain has a hygromycin-resistance gene (Hygromycin B phosphor-transferase), a normal colony morphology, and high virulence in its hosts. The strain AJ3-8 and strain B9 were confirmed as virus-free strains by high-throughput sequencing and RT-PCR detection (data not shown). All strains were cultured on PDA medium at 25°C and then stored at –70°C in 25% glycerol.

### Total RNA Extraction and Sequencing

Total RNA of twenty-two *F. oxysporum* strains were extracted from 1.0 g of mycelium using an RNAiso Plus Kit (TaKaRa, Dalian, China) following the manufacturer’s instructions. Next, total RNA was purified using an RNAClean XP Kit (Cat A63987, Bechman Coulter, Inc., Brea, CA, United States) and RNase-Free DNase set (Cat79254, QIAGEN, GmBH, Hilden, Germany), and rRNA was depleted by a Ribo-ZeroTm rRNA Removal Kit (Illumina, San Diego, CA, United States). Finally, the qualified samples were mixed into one sample and used for pair-end sequencing on an Illumina HiSeq 2500 platform at Shanghai Bohao Biotechnology Co., Ltd. One sequencing library was constructed by the qualified total RNA of *F. oxysporum* strains. The raw reads were filtered base on default parameters, 1 × 10^8^ bp clean reads were obtained and mapped against genome sequence of *F. oxysporum* using Bowtie (1.0) software. Then, unmapped reads were assembled *de novo* using CLC Genomics Workbench (version: 6.0.4) with scaffolding contig algorithm, word-size = 45, and minimum contig length ≥ 200. Consequently, 44,679 contigs were achieved. After Blasted by the non-redundant protein sequences (nr) database in NCBI^1^, 12 contigs which represented partial genome segments of “virus” or “viral” were retrieved. Finally, the contig 1028 that was identical or complementary to mymonavirus genomic sequences were extracted and subjected to further analysis.

### RT-PCR Detection and RACE

The cDNA of each *F. oxysporum* strains were synthesized using a PrimerScript™ 1st Strand cDNA synthesis Kit (TaKaRa, Dalian, China) following the manufacturer’s instructions. The occurrence of putative virus sequence in the *F. oxysporum* strains included in the RNA-Seq sample was investigated using RT-PCR with specific primers designed based on the assembled contig 1028. The RT-PCR results suggested that contig 1028 is the sole viral agent of the LJ3-3 strain (data not shown). The 5′- and 3′-terminal sequences of the FoMyV1 genome were completed using a SMARTer RACE Amplification Kit (Clontech, Mountain View, CA, United States) following the manufacturer’s instructions using gene-specific primers (GSPs). GSP-1028F1 (GAGCAAGAACATAGATTCACCT) and GSP-1028F2 (TGGTTGTGGAGAAATGGGGCTGGTA) were used as the inner and outer primers, respectively, for 3′-RACE. GSP-1028R1 (5′-CTGGCTGGTTTGGTAGGG-3′) and GSP-1028R2 (5′-CTTCGTCGTCTGCCCAAT-3′) were used as the inner and outer primers, respectively, for 5′-RACE. Meanwhile, seven pairs of specific primers were designed to verify the almost full length of FoMyV1 by RT-PCR (Supplementary Table 1 and Supplementary Figure 1). All RT-PCR products of expected size were purified and cloned into the pMD19-T vector (TaKaRa, Dalian, China) and introduced into *Escherichia coli* Trelief 5α (TSINGKE Biotech, Zhengzhou, China) by transformation. At least three recombinant clones were sent to TSINGKE Biotech for sequencing. In addition, the DNA of strain LJ3-3 was used as a template to examine whether the viral sequence was integrated into the host. Moreover, 143 *F. oxysporum* strains from five counties or cities in Henan province of China were used to test for the presence of FoMyV1.

### Sequence and Phylogenetic Analysis

The putative open reading frames (ORFs) of FoMyV1 were deduced using the ORF Finder program on the website of the National Center for Biotechnology Information (NCBI).^2^ Homologous sequences were searched for full-length cDNA sequences and deduced polypeptides of FoMyV1 in the NCBI database using BlastN and BlastP, respectively. A search for the predicted domains present in the polypeptide sequence was conducted using the Conserved Domain Database (CDD).^3^ Multiple sequence alignments of the RdRp sequences were performed using DNAMAN (Version 9) and ClustalX (Version 2.0) (Thompson et al., 1997). A phylogenetic tree was constructed using the maximum-likelihood (ML) method in MEGA-X (Version 10.1.8) with 1,000 bootstrap replicates (Kumar et al., 2018).

### Virus Transmission Assay

To investigate the vertical transmissibility of FoMyV1, 40 single-conidium isolates were obtained from parental strain LJ3-3. Then, the presence of FoMyV1 was determined using RT-PCR with the primer pair ct1028RT-F2/R2 (Supplementary Table 1), which was designed to amplify a 746-bp product.

The pairing-culture technique (Wu et al., 2007; Zhang and Nuss, 2008) was used to investigate the horizontal transmissibility of FoMyV1 between *F. oxysporum* strains. In the contact culture in each plate (9 cm in diameter), the strain LJ3-3 served as the donor, whereas the strain B9 (a hygromycin-resistance-gene transformant of *F. oxysporum*) served as the recipient. The mycelial agar plugs of two strains were cultured at a distance of 2 cm in PDA medium. After incubation of the contact cultures at 25°C for 8 days, three mycelial derivative isolates were obtained from three colonies of the recipient strain in the contact cultures. Then, derivative isolates were cultured in hygromycin-resistance (50 mg/ml) PDA medium three times. Finally, the primer pair ct1028RTF7/F7 with a 1,024-bp amplicon was used to verify the presence of FoMyV1 in the derivative isolates. Two derivative isolates of B9 (B9-VI) infected by FoMyV1 both contained the mycovirus FoMyV1 (Supplementary Figure 2). Compared with strain B9, the colony of B9-VI was irregular and the aerial hyphae were rare.

### Biological Characterization and Virulence Assay

To assess the effects of FoMyV1 on its host biological characteristics and plant pathogenicity, two isogenic strains B9-VI (virus-infected) and B9 (virus-free) were used. Each strain was individually tested for mycelial growth rate (PDA, 25°C) and conidium production. Five mycelial plugs were inoculated into 100 ml carboxymethylcellulose sodium (CMC) fluid medium and cultured for 4 days (28°C, 180 rpm). Then, the mycelium solution was filtered through two layers of sterile gauze and the precipitate was resuspended with 50 ml sterile water. Finally, the concentration of conidium was counted using a blood counting plate and the conidium production was calculated. Tobacco cultivar ‘Zhongyan 100’ and *Nicotiana benthamiana* were grown to the third or fourth leaf stage and then individuals with the same growth status were selected. The roots were injured and then tobacco seedlings were transplanted into new pots (9 cm × 7 cm × 6 cm, top width × bottom width × height), inoculated with 30 ml spore suspension (1 × 10^7^ml^–1^), and cultured at 25°C under fluorescent light (16 h light/8 h dark). One month later, investigate the disease incidence, severity, index, and phenotypic values of plants. Disease incidence was defined as the percentage of infected plants, and disease severity was rated on a scale of 0–9 as follows: level 0, no symptoms; level 1, the plant growth is basically normal or slightly dwarfing, a few roots are necrotic and dark brown, middle and lower leaves are chlorosis or discoloration; level 3, the disease plants are 1/4–1/3 lower than the healthy ones, half of the roots are necrotic and black, 1/2–2/3 of the leaves are wilting; level 5, the disease plants are 1/3–1/2 lower than the healthy ones, most of the roots are necrotic and black, more than 2/3 of the leaves are wilting, tip and margin of the middle and lower leaf are slightly withered; level 7, the disease plants are more than 1/2 lower than the healthy ones, all of the leaves are wilting, all of the roots are necrotic and black, and the secondary roots near the surface were obviously damaged; level 9, plant is dead. Pathogen was re-isolated from seedlings inoculated with strain B9-VI and detected to carry fungal virus FoMyV1. The assay treatments were repeated three times with three seedlings each. A one-way factorial analysis of variance (ANOVA) (SAS Institute, Cary, NC, United States, Version 8.0, 1999) was used to determine the differences in growth rate, conidial production, phenotypic values, and disease index of each strain.

## Results

### Biological Characteristics of *Fusarium oxysporum* Strain LJ3-3

**FIGURE 1:**
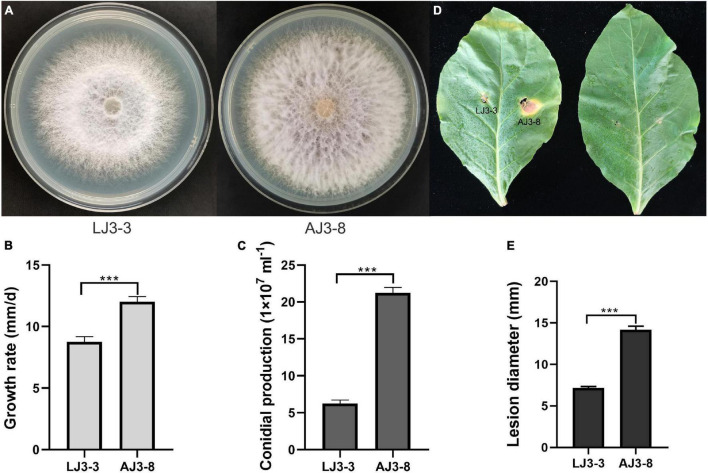
The comparison of different biological characteristic of strain LJ3-3 and AJ3-8. Culture morphology (25°C, 10 days) **(A)** and pathogenicity assay (28°C, 13 days) **(D)** of *Fusarium oxysporum* strain LJ3-3 and AJ3-8 on PDA medium and detached Zhongyan 100 leaves, respectively. Comparison of radial mycelial growth rate (25°C) on PDA **(B)**, conidia production (28°C, 180 rpm, right) in CMC **(C)**, and lesion diameter (28°C, 13 days) on detached Zhongyan 100 leaves **(E)** of strain LJ3-3 and AJ3-8, respectively. “***” indicates a significantly different (*p* < 0.05) between strain LJ3-3 and AJ3-8 in radial mycelial growth rate, conidia production, and lesion diameter.

Based on RT-PCR and EF-1α, RPB1, and RPB2 sequencing, we identified strain LJ3-3 as *F. oxysporum* (Supplementary Table 3). The sample also contained the (–)ssRNA mycovirus FoMyV1 (Supplementary Figure 2). We cultured *F. oxysporum* strain LJ3-3 at 25°C on PDA for 10 days to observe its morphology (Figure 1A). The average radial mycelial growth of LJ3-3 was 8.75 mm/day, which was significantly (*p* < 0.05) slower than that of the virus-free strain AJ3-8 (12.00 mm/day) (Figure 1B). The average conidial production of LJ3-3 was 6.45 × 10^7^ml^–1^, which was also significantly (*p* < 0.05) lower than that of strain AJ3-8 (21.50 × 10^7^ml^–1^; Figure 1C). In the virulence assay using Zhongyan 100 leaves, the average lesion diameter (7 mm) caused by strain LJ3-3 was significantly (*p* < 0.05) smaller than that caused by strain AJ3-8 (14 mm; Figures 1D,E). For comparation, strain LJ3-3 infected with FoMyV1 showed slow growth rate, low conidial production, and weak virulent.

### Genome Analysis of *Fusarium oxysporum* Mymonavirus 1

**FIGURE 2:**
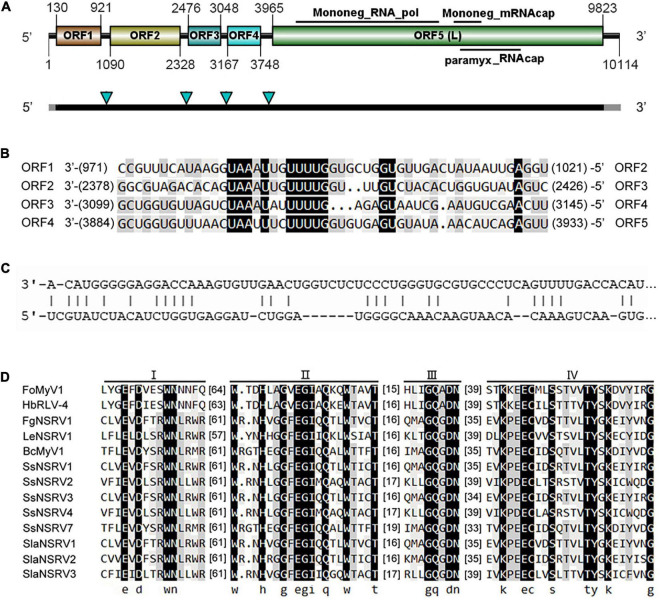
Genome organization of a mymonavirus from the *F. oxysporum* strain LJ3-3. **(A)** Schematic diagram of the genome organization of *Fusarium oxysporum* mymonavirus 1 (FoMyV1). FoMyV1 shows the presence of five ORFs. The black bars indicate the coding regions, and the gray bars represent the untranslated regions on the genome of FoMyV1. The four green arrowheads point out the location of the putative gene junction sequence. **(B)** Comparison of putative gene-junctions between ORFs in the FoMyV1, alignment of the putative junction sequences are shown in the 3′–5′ orientation. **(C)** Complementarity between the 3′- and 5′-terminal sequences of FoMyV1 genomic RNA strand. **(D)** Multiple alignments of the amino acid sequences of RdRp in the protein L encoded by FoMyV1 and other (–)ss RNA viruses. The abbreviations of virus names are listed in Supplementary Table 4.

The complete genome sequence of FoMyV1 (GenBank accession no. OM049502) was 10,114 nt, with a GC% content of 47%, possessing five non-overlapping ORFs (ORF1–5) and two untranslated regions (UTR) of 129 and 291 nt at the 5′- and 3′-termini, respectively (Figure 2A). ORF1–ORF5 encode proteins 263, 412, 190, 193, and 1,952 amino acids (aa) in length, located in the reading frames +1, +1, +1, +2, and + 2, respectively. The conserved motif search showed that ORF5 contained a *Mononegavirales* RNA-dependent RNA polymerase domain (pfam00946; aa location 142–980; e-value 1.61e-112), *Mononegavirales* mRNA-capping region V (pfam14318; aa location 1,077–1,231; e-value 3.43e-12), and paramyxovirus_RNAcap (TIGR04198; aa location 1,114–1,462; e-value 3.82e-06; Figure 2A). We did not find any conserved domains in the other four ORFs. BlastP analysis showed that the putative protein L encoded by ORF5 of FoMyV1 was similar to the RdRp of Hubei rhabdo-like virus 4 (HbRLV4) with 65% identity (Shi et al., 2016). In addition, the putative protein L also showed 26–36% identity with the RdRp encoded by other mymonaviruses in the family *Mymonaviridae* (Supplementary Table 3). The proteins encoded by ORF1 and ORF2 were similar to the hypothetical proteins 1 and 2 of HbRLV4, with 43 and 67% identity, respectively (Table 1). However, the putative protein encoded by ORF3 and ORF4 of FoMyV1 was not significantly similar to any other protein in the search to characterize. In addition, the semi-conserved AU-rich sequences are finds in the putative untranslated sequences between ORFs in the FoMyV1 genome (Figure 2B). The putative gene-junction sequence of ORF1/2 and ORF2/3 (viral RNA strand, 3′-UAAAUUGUUUUG-5′) was identical to those of HbRLV4. We also found several complementary nucleotides near the end of the FoMyV1 genome sequence (Figure 2C). We identified four conserved motifs (I–IV) from *Mononegavirales* in protein L encoded by FoMyV1 ORF5 (Figure 2D). Meanwhile, FoMyV1 was not detected in the DNA template of LJ3-3 strain (Supplementary Figure 3). In summary, FoMyV1 genome structural characteristics were consistent with the typical characteristics of members in order *Mononegavirales*, and belong to the family *Mymonaviridae*.

**TABLE 1:** Information about first blastp hit for each predicted protein encoded by *Fusarium oxysporum* mymonavirus 1.

Virus	ORF	Blastp First Hit	Query cover	E value	Per/Ident	Accession
*Fusarium oxysporum* mymonavirus 1	ORF1	hypothetical protein 1 [Hubei rhabdo-like virus 4]	100%	1e-54	43.35%	YP_009336593.1
	ORF2	hypothetical protein 2 [Hubei rhabdo-like virus 4)]	99%	0	67.31%	YP_009336594.1
	ORF3	No significant similarity found	/	/	/	/
	ORF4	No significant similarity found	/	/	/	/
	ORF5	RNA-dependent RNA polymerase [Hubei rhabdo-like virus 4]	99%	0	64.94%	YP_009336595.1

*Blastp search was conducted using NCBI-BLAST.*

### Phylogenetic Analysis of *Fusarium oxysporum* Mymonavirus 1 and Other Mymonaviruses

**FIGURE 3:**
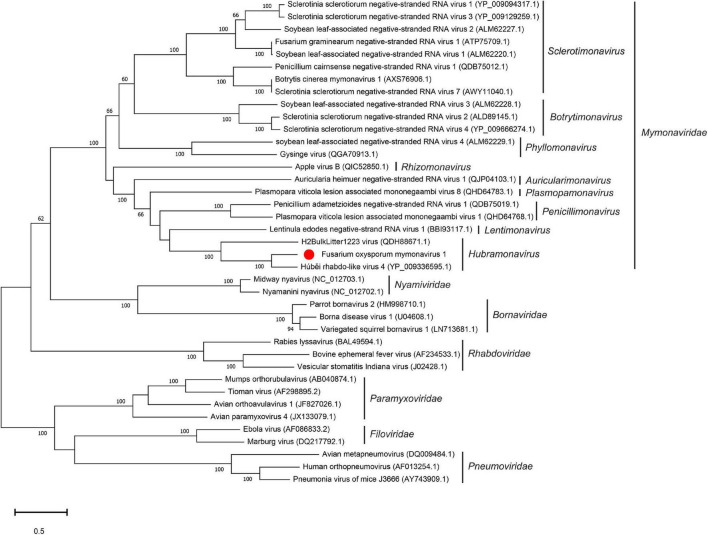
Phylogenetic analysis of FoMyV1 (marked with a red dot) and other related (–)ss RNA viruses. The phylogenetic tree was generated by the maximum-likelihood method (1,000 bootstrap replicates) based on the amino acid sequences of the RdRp domains using MEGA-X.

To examine the relationship between FoMyV1 and other mymonaviruses (Supplementary Table 4), we performed a maximum-likelihood phylogenetic analysis based on the amino acid sequences of the L protein of FoMyV1 and 38 other (–)ssRNA viruses, including representative members of six families in order *Mononegavirales* (*Nyamiviridae*, *Bornaviridae*, *Rhabdoriridae*, *Paramyxoviridae*, *Filoviridae*, and *Pneumoviridae*), and representative strains of nine genus in family *Mymonaviridae*. FoMyV1 clustered with HbRLV4 and H2BulkLitter 1223 virus (Starr et al., 2019) to form a distinct clade with a bootstrap support value of 100%, indicating a close evolutionary relationship. These three viruses belong to the genus *Hubramonavirus* and clustered with 22 other mymonaviruses, forming a large independent clade of family *Mymonaviridae* (Figure 3). The other 17 (–)ssRNA viruses also formed corresponding viral family clades. These results confirm that FoMyV1 is a novel member of the genus *Hubramonavirus*, family *Mymonaviridae*.

### Horizontal Transmission of *Fusarium oxysporum* Mymonavirus 1 between *Fusarium oxysporum* Strains

**FIGURE 4:**
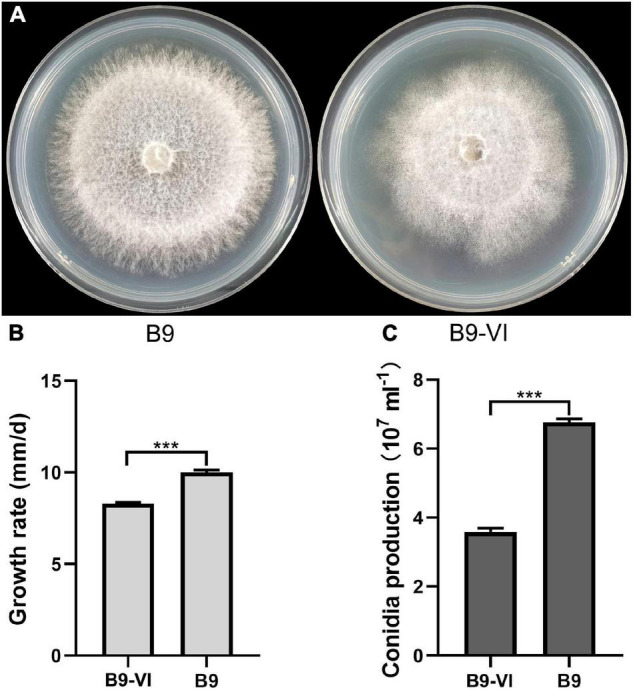
The comparison of different biological characteristic of strain B9 and B9-VI. **(A)** Culture morphology (25°C, 10 days) of *Fusarium oxysporum* strain B9 and B9-VI on PDA medium. **(B,C)** Comparison of radial mycelial growth rate **(B)** on PDA (25°C) and conidia production **(C)** in CMC (28°C, 180 rpm) of strain B9-VI and B9, respectively. “***” indicates a significantly different (*p* < 0.05) between strain B9 and B9-VI in both radial mycelial growth rate and conidia production.

We used *F. oxysporum* strain B9 as a recipient for horizontal transmission of FoMyV1. We obtained one mycelial derivative isolate—B9-VI—from one B9 recipient colony in the two contact cultures of LJ3-3/B9 (Figure 4A). The average growth rate of B9-VI was 8.29 mm/day, which was significantly slower than that of B9 (10.00 mm/day; Figure 4B). Similarly, the conidium production of B9-VI was 3.35 × 10^7^ ml^–1^, significantly lower than that of B9 (6.76 × 10^7^ ml^–1^; Figure 4C). Furthermore, RT-PCR indicated that FoMyV1 was successfully transmitted from LJ3-3 to the virus-free strain B9Hyg*^R^* (Supplementary Figure 2). In summary, colony morphology, growth rate, and conidium production were significantly affected by the FoMyV1 infection (Figure 4).

### Transmission of *Fusarium oxysporum* Mymonavirus 1 to Conidium Progeny

To determine the frequency of FoMyV1 transmission by conidium progeny in the laboratory, we obtained 40 single-conidium isolates from parent strain LJ3-3 and tested for FoMyV1 using RT-PCR. All the single-conidium isolates were FoMyV1 positive (Supplementary Figure 4). The average growth rate of the isolates was 6.6–9.6 mm/day in PDA plate. There were no significant differences in growth rate among the 40 conidium progeny (*p* < 0.05).

### Effect of *Fusarium oxysporum* Mymonavirus 1 on Host Virulence

**FIGURE 5:**
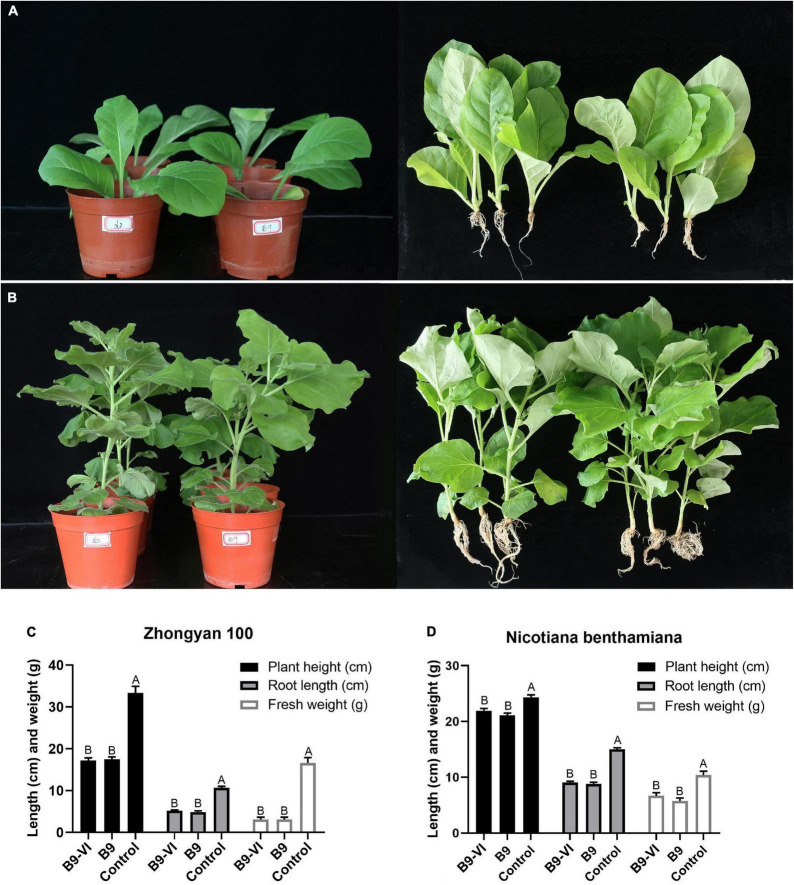
Comparison of pathogenicity between *F. oxysporum* strain B9-VI (virus infect) and B9 (virus-free) on the tobacco cultivar ‘Zhongyan 100’ and *N. benthamiana*. **(A)** Strain B9-VI and B9 inoculated tobacco cultivar ‘Zhongyan 100’ were grown in pots for 31 days (left), and comparison of growth status of plants inoculated with strain B9-VI and B9 (right). **(B)** Strain B9-VI and B9 inoculated tobacco cultivar *N. benthamiana* were grown in pots for 31 days (left), and comparison of growth status of plants inoculated with strain B9-VI and B9 (right). **(C,D)** Average plant height, root length, and fresh weight of two tobacco cultivars inoculated with strain B9-VI and B9. Strain B9-VI infects by FoMyV1, strain B9 is the mycovirus-free. The “control” in panels **(C,D)** represents plants that have not been inoculated with any pathogens.

To study the effect of FoMyV1 on the virulence of its fungal host, we evaluated the pathogenicity of two isogenic strains, B9-VI (virus-infected) and B9 (virus-free), in two different tobacco cultivars. The disease index of B9-VI and B9 in tobacco cultivar ‘Zhongyan 100’ was 35.80 and 38.27, respectively, and those in *N. benthamiana* were 8.64 and 9.87, respectively (Supplementary Table 5). There were no significant differences in disease index between the two strains on either cultivar (*p* < 0.05). Furthermore, there were no significant differences (*p* < 0.05) in plant height, fresh weight, or root length between plants inoculated with the two strains in either plant cultivar (Figure 5). At the same time, we re-isolated the pathogen from the root of B9-VI infected plants for FoMyV1 detection. The two re-isolated strains were detected as FoMyV1 positive (Supplementary Figure 5). These results suggest that FoMyV1 does not alter the pathogenicity of its host.

### Incidence of *Fusarium oxysporum* Mymonavirus 1

To investigate the incidence of FoMyV1 in Henan Province, 143 *F. oxysporum* strains (Supplementary Table 6) were tested for the presence of FoMyV1 using RT-PCR with primer pair ct1028RT-F7/R7 (Supplementary Table 1). The result showed that only two strains harbored FoMyV1 in the tested 143 *F. oxysporum* strains (Supplementary Figure 2), and the incidence of FoMyV1 was only 1%. The FoMyV1 infected strain LJ3-3 and LJ4-1 were both collected from same location (Xuchang city, Henan Province, China).

## Discussion

In this work, we identified and characterized an (–)ssRNA mycovirus found in a strain of *Fusarium oxysporum*, which was isolated from capsicum. Based on homology BlastX searches, genome organization comparison, and phylogenetic analysis, we propose that this (–)ssRNA virus is a novel member of the genus *Hubramonavirus* in the family *Mymonaviridae*. We name it *Fusarium oxysporum* mymonavirus 1 (FoMyV1).

*Fusarium oxysporum* is a plant pathogenic fungus that is distributed worldwide. It causes vascular diseases and fusarium root rot in many economically important crops, leading to serious economic losses (Michielse and Rep, 2010). The known mycoviruses associated with *F. oxysporum* are limited and belong to the families *Chrysoviridae*, *Hypoviridae*, *Mitoviridae*, *Polymycoviridae*, *Botourmiaviridae*, and *Alternaviridae*. Of these reported mycoviruses, only FodV1 and FoOuLV1 are hypovirulent and could be used as biological control agents (Lemus-Minor et al., 2019; Zhao et al., 2020). The hypovirus FodHV2 does not affect the vegetative growth, conidiation, or the virulence of its fungal host (Torres-Trenas et al., 2020). In contrast, FoMyV1 infection reduced the vegetative growth and conidial production of its host, but did not affect the pathogenicity. FoMyV1 is also stable in its conidium progeny. Therefore, FoMyV1 may interact with the fungus to modulate its vegetative growth and conidia production without affecting its virulence. Then, the reason for this need to be further studied.

Liu et al. (2014) reported the genome structure, virion morphology, transcription strategy, and infectivity of the first known (–)ssRNA mycovirus, Sclerotinia sclerotiorum negative-stranded RNA virus 1 (SsNSRV1), which is most closely related to *Bornaviridae* and *Nyamiviridae* in the order *Mononegavirales*. With the development of high-throughput sequencing technology, many more mycoviruses have been identified and characterized, leading to the establishment of a new family of viruses, the *Mymonaviridae*, which contains nine genera and 32 species (Jiāng et al., 2019). SsNSRV1 belongs to the genus *Sclerotimonavirus* in the family *Mymonaviridae*. The typical mymonavirus genome contains five or six major non-overlapping ORFs that are arranged linearly. ORF II and ORF V encode the nucleoprotein and RdRp, respectively. Similarly, FoMyV1 and HbRLV4 both contain five ORFs, the ORF2 and ORF5 encodes putative nucleoprotein and L protein, respectively. However, the remaining three ORFs encode proteins do not match known viral proteins. In contrast to HbRLV4, FoMyV1’ L protein also contains one domain paramyxovirus_RNAcapping region (TIGR04198). The function of this domain is capping of mRNA, which requires RNA triphosphatase and guanylyl transferase activities, demonstrated for rinderpest virus L protein (Gopinath and Shaila, 2009). The GC content of the FoMyV1 RNA is 47%, slightly lower than that of HbRLV4 (48%), and slightly higher than that of SsNSRV-1 (39%). The gene-junction sequences are ubiquitous in the mononegaviral genomes and are important for transcriptional regulation (Conzelmann, 1998). The putative gene-junction sequence of ORF1/2 and ORF2/3 is identical to those of HbRLV4, but not identical to those of other mymonaviruses (Liu et al., 2014; Lin et al., 2019). Comparison of the complete nucleotide and amino acid sequences of FoMyV1 and HbRLV4 showed a high similarity (58 and 65%, respectively). However, the amino acid sequence of FoMyV1 ORF1 and ORF2 were only similar to those of HbRLV4 hypothetical protein 1 and hypothetical protein 2, with 43 and 67% identity, respectively. Moreover, a phylogenetic analysis showed that FoMyV1 formed a tight cluster with HbRLV4 (derived from an arthropod mix) and then clustered with H2BulkLitter1223 virus (derived from grassland soil), forming an independent clade of *Hubramonavirus* in family *Mymonaviridae* with a bootstrap support value of 100%. In brief, we characterized a novel mymonavirus, FoMyV1, in the genus Hubramonavirus that could infect fungi in nature. This is the first reported (–)ssRNA mycovirus associated with *F. oxysporum*.

Mycoviruses infect all the major taxa of fungi. In general, mycoviruses are transmitted horizontally via anastomosis of vegetatively compatible strains of the same species and vertically by disseminating sexual or asexual spores (Ghabrial et al., 2015). However, the fungal DNA virus Sclerotinia sclerotiorum hypovirulence-associated DNA virus 1 can be transmitted through insect vectors, which extends our traditional understanding fungal virus transmission mechanisms (Liu et al., 2016). Sclerotinia sclerotiorum mycoreovirus 4 (SsMYRV4) can overcome the hurdle of vegetatively incompatible groups via suppressing non-self-recognition by the fungus host. Therefore, SsMYRV4 infection facilitates the horizontal transmission of other mycoviruses across vegetatively incompatible groups (Wu et al., 2017). Several mycoviruses, such as Cryphonectria hypovirus 1, Cryphonectria hypovirus 4, and Rosellinia necatrix mycoreovirus 3 (RnMyV3), encode RNA silencing suppressor proteins (RSS) to escape the host RNA silencing for horizontal transmission (Segers et al., 2006; Yaegashi et al., 2013; Aulia et al., 2021). In contrast, Mycoreovirus 1, which originated from *Cryphonectria parasitica*, can induce silencing genes dicer-like 2 (*dcl*2) and argonaute-like 2 (*agl*2), which activate the antiviral RNA silencing of the host and constrain the infection of other mycoviruses (Chiba and Suzuki, 2015; Yang et al., 2021). In view of the biological characteristics of FoMyV1 infected its host, it can be used as a typical material for the studying the molecular mechanism of fungus-virus interaction.

More than 80% of plant diseases are caused by fungal pathogens that cause yield reduction and mildewing in crop plants. The most environmentally friendly ways to control diseases are the development of resistant varieties and the use of beneficial microbes (Fravel, 2005). For example, the mycovirus CHV1 has been successfully used as a biological control agent against chestnut blight (*Cryphonectria parasitica*) (Anagnostakis, 1982). One biological control mechanism of CHV1 is that it encodes and utilizes RNA silencing suppressors against the host defense (Segers et al., 2006). An ubiquitin-like protein, ATG8, is a key element of the autophagy pathway (Klionsky et al., 2016). Moreover, CHV1 infection can regulate a homologous gene *Cpatg8* that is required for virulence and development of chestnut blight fungus, as well as accumulation of viral dsRNA replicative form in the fungus (Shi et al., 2019). Fungal DNA virus SsHADV1 could infect a mycophagous insect (*Lycoriella ingenua*), and use it as a transmission vector (Liu et al., 2016). As we all know, the nutritional incompatibility of fungi is one of the limiting factors for horizontal transmission of mycovirus (Ghabrial et al., 2015). The finding implies that mycoviruses could be transmitted via insects, and also provided a new idea on how to use fungal virus to control fungal plant diseases. Furthermore, the SsHADV1-infected *S. sclerotiorum* strain DT-8 can grow endophytically in monocots, protecting against fungal disease (Tian et al., 2020). In summary, diseases could be controlled by hypovirulence-associated mycoviruses.

*Fusarium oxysporum* is a fungus with a wide range of hosts. It includes pathogenic and non-pathogenic strains, and several non-pathogenic strains have been widely applied as biocontrol agents (Gordon and Martyn, 1997; Fravel and Alabouvette, 2003). For example, an endophytic *F. oxysporum* strain was found to induce systemic resistance against nematode (*Radopholus similis*) infection in banana plants (Vu et al., 2006). There is abundant mycovirus diversity in *Fusarium*, and some mycoviruses are associated with hypovirulence (Li et al., 2019; Zhao et al., 2020). Our expectation is to apply these hypovirulence-associated mycoviruses to control Fusarium disease. It may be associated with non-pathogenic or endophytic traits of *F. oxysporum* strains. However, this requires further study.

## Data Availability Statement

The datasets presented in this study can be found in online repositories. The names of the repository/repositories and accession number(s) can be found below: NCBI database, accession number OM049502.

## Author Contributions

JW designed the research. CL, PS, XL, RS, YN, and HZ collected the materials. JW, CL, RQ, and PS performed the experiments. JW, SL, and HL wrote the first draft of the manuscript. All authors critically reviewed the manuscript and approved the final submission.

## Conflict of Interest

RS was employed by Tobacco Company of Henan Province. The remaining authors declare that the research was conducted in the absence of any commercial or financial relationships that could be construed as a potential conflict of interest.

## Publisher’s Note

All claims expressed in this article are solely those of the authors and do not necessarily represent those of their affiliated organizations, or those of the publisher, the editors and the reviewers. Any product that may be evaluated in this article, or claim that may be made by its manufacturer, is not guaranteed or endorsed by the publisher.

## Footnotes

1
https://blast.ncbi.nlm.nih.gov/Blast.cgi?PROGRAM=blastx&PAGE_TYPE= BlastSearch&LINK_LOC=blasthome

2
https://www.ncbi.nlm.nih.gov/orffinder/

3
https://www.ncbi.nlm.nih.gov/cdd

## Supplementary Material

Supplementary Figure 1 Agarose gel electrophoresis of Partial FoMyV1 genome validated by RT-PCR with seven primers.

Supplementary Figure 2 RT-PCR detection of FoMyV1 in strain LJ3-3, LJ4-1, and derivative strain B9-VI.

Supplementary Figure 3 FoMyV1 detection by DNA template of strain LJ3-3.

Supplementary Figure 4 RT-PCR detection of FoMyV1 in the conidia progeny of strain LJ3-3.

Supplementary Figure 5 RT-PCR detection of FoMyV1 from re-isolated strain in the root of B9-VI infected plant.
