# Supplementary material for: Molecular and Biological Characterization of the First Mymonavirus Identified in Fusarium oxysporum
Source: Front Microbiol. 2022 Apr 21;13:870204. doi: 10.3389/fmicb.2022.870204 (PMC9069137; doi:10.3389/fmicb.2022.870204)
Supplement: Supplementary Figure 1 — Agarose gel electrophoresis of Partial FoMyV1 genome validated by RT-PCR with seven primers. [file Data_Sheet_1.zip › Table S1.DOCX]

| Name | Primer（5’-3’） | Location | Product (bp) | Tm °C |
| --- | --- | --- | --- | --- |
| ct1028RT-F1 | GTCTCTCCCTGGGTGCGTG | 29-800 | 771 | 60 |
| ct1028RT-R1 | GACTTTGCTGGTGCCTTGGG |  |  |  |
| ct1028RT-F2 | CTTTGCTGCTGCTGCTACACAG | 224-970 | 746 | 59 |
| ct1028RT-R2 | GGGAATGCTTGGACAGGCTCA |  |  |  |
| ct1028RT-F3 | CTCGCAATAGGACACTGCCC | 4841-5658 | 817 | 58 |
| ct1028RT-R3 | GCTTGGAAATTGTTGTTCCAGCTTTCC |  |  |  |
| ct1028RT-F4 | GAGATGACATGGGATCCAGCCG | 5572-6397 | 825 | 58 |
| ct1028RT-R4 | CGATGTGCTCCCCAAGTAACTAGT |  |  |  |
| ct1028RT-F5 | ATGAGATGGTCTCCCATTCCTCTT | 8074-8800 | 726 | 57 |
| ct1028RT-R5 | GTCATGGTTGGAGATTCCAATCATCC |  |  |  |
| ct1028RT-F6 | ACTGGTCTCTGTCACTGGATCAACA | 8743-9431 | 723 | 59 |
| ct1028RT-R6 | TGGGGACAGTGGTAGTTACCGGA |  |  |  |
| ct1028RT-F7 | TATGACGGTGAAGATGGGT | 3624-4666 | 1024 | 52 |
| ct1028RT-R7 | ACCAACATTCCCATACCTT |  |  |  |

Table S1. Primers used to identify the almost complete genome of FoMyV1 by RT-PCR.
