# Supplementary material for: Molecular and Biological Characterization of the First Mymonavirus Identified in Fusarium oxysporum
Source: Front Microbiol. 2022 Apr 21;13:870204. doi: 10.3389/fmicb.2022.870204 (PMC9069137; doi:10.3389/fmicb.2022.870204)
Supplement: Supplementary Figure 1 — Agarose gel electrophoresis of Partial FoMyV1 genome validated by RT-PCR with seven primers. [file Data_Sheet_1.zip › Table S2.DOCX]

Supplementary Table S2: The results of target genes from strain LJ3-3 searched in a dedicated site for fusarium. ([Cyber-infrastructure for Fusarium (fusariumdb.org)](http://www.fusariumdb.org/))

| Gene | Fusarium ID/  Accession Number | Fusarium spp. | Identity (%) |
| --- | --- | --- | --- |
| RPB1 | FD_02182 | F. oxysporum species complex 186 NRRL38302 | 99.62 |
|  | FD_02003 | F. oxysporum species complex 63 NRRL34936 | 99.33 |
| EF-1a | FD_00321 | F. oxysporum species NRRL32885 | 99.69 |
|  | FD_00532 | F. oxysporum species complex 63 NRRL36570 | 99.69 |
| RPB2 | FD_02182 | F. oxysporum species complex 186 NRRL38302 | 99.44 |
|  | FD_00120 | F. oxysporum species complex 1 NRRL26360 | 99.43 |
| ITS | MT967363.1 | F. oxysporum isolate ORTO924 | 99.61 |
|  | MW513779.1 | F. oxysporum strain SMSJG02 | 99.22 |
