# Supplementary material for: Molecular and Biological Characterization of the First Mymonavirus Identified in Fusarium oxysporum
Source: Front Microbiol. 2022 Apr 21;13:870204. doi: 10.3389/fmicb.2022.870204 (PMC9069137; doi:10.3389/fmicb.2022.870204)
Supplement: Supplementary Figure 1 — Agarose gel electrophoresis of Partial FoMyV1 genome validated by RT-PCR with seven primers. [file Data_Sheet_1.zip › Table S3.DOCX]

Supplementary Table S3. BlastP results of RNA-dependent RNA polymerase encoded by ORF5 of Fusarium oxysporum mymonavirus 1.

| Genus | Virus name | Query Cover (%) | aa Identity (%) | E value | Accession no. |
| --- | --- | --- | --- | --- | --- |
| *Hubramonavirus* | Hubei rhabdo-like virus 4 | 99 | 64.94 | 0.0 | YP_009336595.1 |
|  | H2BulkLitter1223 virus | 98 | 36.37 | 0.0 | QDH88671.1 |
| *Penicillimonavirus* | Penicillium adametzioides negative-stranded RNA virus 1 | 63 | 34.03 | 0.0 | QDB75019.1 |
|  | Plasmopara viticola lesion associated mononegaambi virus 6 | 83 | 30.66 | 0.0 | QHD64777.1 |
| *Lentimonavirus* | Lentinula edodes negative-strand RNA virus 1 | 84 | 30.83 | 0.0 | BBI93117.1 |
| *Plasmopamonavirus* | Plasmopara viticola lesion associated mononegaambi virus 8 | 97 | 29.46 | 0.0 | QHD64783.1 |
| *Sclerotimonavirus* | Sclerotinia sclerotiorum negative-stranded RNA virus 1 | 59 | 29 | 4e-114 | YP_009094317.1 |
|  | Plasmopara viticola lesion associated mymonavirus 1 | 78 | 26.05 | 5e-113 | QHD64779.1 |
| *Botrytimonavirus* | Botrytis cinerea negative-stranded RNA virus 7 | 65 | 29.83 | 3e-51 | QKW91270.1 |
| *Auricularimonavirus* | Auricularia heimuer negative-stranded RNA virus 1 | 71 | 28.97 | 2e-150 | QJP04103.1 |
