# Supplementary material for: Molecular and Biological Characterization of the First Mymonavirus Identified in Fusarium oxysporum
Source: Front Microbiol. 2022 Apr 21;13:870204. doi: 10.3389/fmicb.2022.870204 (PMC9069137; doi:10.3389/fmicb.2022.870204)
Supplement: Supplementary Figure 1 — Agarose gel electrophoresis of Partial FoMyV1 genome validated by RT-PCR with seven primers. [file Data_Sheet_1.zip › Table S4.DOCX]

Supplementary Table S4. The detailed information of selected mononegaviruses used in phylogenetic analysis.

| **Family** | **Genus** | **Virus Name** | **Acession Number** |
| --- | --- | --- | --- |
| *Mymonaviridae* | *Sclerotimonavirus* | Sclerotinia sclerotiorum negative-stranded RNA virus 1 | YP_009094317.1 |
|  |  | Sclerotinia sclerotiorum negative-stranded RNA virus 3 | YP_009129259.1 |
|  |  | Soybean leaf-associated negative-stranded RNA virus 1 | ALM62220.1 |
|  |  | Fusarium graminearum negative-stranded RNA virus 1 | ATP75709.1 |
|  |  | Soybean leaf-associated negative-stranded RNA virus 2 | ALM62227.1 |
|  |  | Sclerotinia sclerotiorum negative-stranded RNA virus 7 | AWY11040.1 |
|  |  | Botrytis cinerea mymonavirus 1 | AXS76906.1 |
|  |  | Penicillium cairnsense negative-stranded RNA virus 1 | QDB75012.1 |
|  | *Botrytimonavirus* | Soybean leaf-associated negative-stranded RNA virus 3 | ALM62228.1 |
|  |  | Sclerotinia sclerotiorum negative-stranded RNA virus 2 | ALD89145.1 |
|  |  | Sclerotinia sclerotiorum negative-stranded RNA virus 4 | YP_009666274.1 |
|  | *Phyllomonavirus* | soybean leaf-associated negative-stranded RNA virus 4 | ALM62229.1 |
|  |  | Gysinge virus | QGA70913.1 |
|  | *Rhizomonavirus* | apple virus B | QIC52850.1 |
|  | *Auricularimonavirus* | Auricularia heimuer negative-stranded RNA virus 1 | QJP04103.1 |
|  | *Plasmopamonavirus* | Plasmopara viticola lesion associated mononegaambi virus 8 | QHD64783.1 |
|  | *Penicillimonavirus* | Penicillium adametzioides negative-stranded RNA virus 1 | QDB75019.1 |
|  |  | Plasmopara viticola lesion associated mononegaambi virus 1 | QHD64768.1 |
|  | *Lentimonavirus* | Lentinula edodes negative-strand RNA virus 1 | BBI93117.1 |
|  | *Hubramonavirus* | Húběi rhabdo-like virus 4 | YP_009336595.1 |
|  |  | H2BulkLitter1223 virus | QDH88671.1 |
| ***Nyamiviridae*** |  | Midway nyavirus | NC_012703.1 |
|  |  | Nyamanini nyavirus | NC_012702.1 |
| ***Bornaviridae*** |  | Parrot bornavirus 2 | HM998710.1 |
|  |  | Borna disease virus 1 | U04608.1 |
|  |  | Variegated squirrel bornavirus 1 | LN713681.1 |
| ***Rhabdoviridae*** |  | Rabies lyssavirus | BAL49594.1 |
|  |  | Bovine ephemeral fever virus | AF234533.1 |
|  |  | Vesicular stomatitis Indiana virus | J02428.1 |
| ***Paramyxoviridae*** |  | Mumps orthorubulavirus | AB040874.1 |
|  |  | Tioman virus | AF298895.2 |
|  |  | Avian orthoavulavirus 1 | JF827026.1 |
|  |  | Avian paramyxovirus 4 | JX133079.1 |
| ***Filoviridae*** |  | Ebola virus | AF086833.2 |
|  |  | Marburg virus | DQ217792.1 |
| ***Pneumoviridae*** |  | Avian metapneumovirus | DQ009484.1 |
|  |  | Human orthopneumovirus | AF013254.1 |
|  |  | Pneumonia virus of mice J3666 | AY743909.1 |
