# Supplementary material for: Molecular and Biological Characterization of the First Mymonavirus Identified in Fusarium oxysporum
Source: Front Microbiol. 2022 Apr 21;13:870204. doi: 10.3389/fmicb.2022.870204 (PMC9069137; doi:10.3389/fmicb.2022.870204)
Supplement: Supplementary Figure 1 — Agarose gel electrophoresis of Partial FoMyV1 genome validated by RT-PCR with seven primers. [file Data_Sheet_1.zip › Table S5.DOCX]

Supplementary Table S5. The pathogenicity comparison of strain B9-VI and B9 in two different tobacco varieties.

| Isolate | FoMyV1 |  | Disease index | | |
| --- | --- | --- | --- | --- | --- |
|  |  |  | Zhongyan 100 |  | *N. benthamiana* |
| B9-VI | + |  | 35.80 |  | 8.64 |
| B9 | - |  | 38.27 |  | 9.87 |

*+ Means isolate harbored mycovirus FoMyV1, and – means not.*
