# Supplementary material for: Molecular and Biological Characterization of the First Mymonavirus Identified in Fusarium oxysporum
Source: Front Microbiol. 2022 Apr 21;13:870204. doi: 10.3389/fmicb.2022.870204 (PMC9069137; doi:10.3389/fmicb.2022.870204)
Supplement: Supplementary Figure 1 — Agarose gel electrophoresis of Partial FoMyV1 genome validated by RT-PCR with seven primers. [file Data_Sheet_1.zip › Table S6.DOCX]

Supplementary Table S6. The source information of 143 tested strains collected from Henan Province for high-throughput sequencing.

| Location (County/City) | Year | Host | Number |
| --- | --- | --- | --- |
| Xiangxian | 2019, 2020 | Tobacco | 88 |
| Luohe | 2020 | capsicum | 6 |
| Pingdingshang | 2020 | Tobacco | 7 |
| Sanmengxia | 2020 | Tobacco | 42 |
